# Hyperactive Akt1 Signaling Increases Tumor Progression and DNA Repair in Embryonal Rhabdomyosarcoma RD Line and Confers Susceptibility to Glycolysis and Mevalonate Pathway Inhibitors

**DOI:** 10.3390/cells11182859

**Published:** 2022-09-14

**Authors:** Silvia Codenotti, Daniela Zizioli, Luca Mignani, Sara Rezzola, Giovanna Tabellini, Silvia Parolini, Arianna Giacomini, Michela Asperti, Maura Poli, Delia Mandracchia, Marika Vezzoli, Simona Bernardi, Domenico Russo, Stefania Mitola, Eugenio Monti, Luca Triggiani, Davide Tomasini, Stefano Gastaldello, Matteo Cassandri, Rossella Rota, Francesco Marampon, Alessandro Fanzani

**Affiliations:** 1 Department of Molecular and Translational Medicine, University of Brescia, 25123 Brescia, Italy; 2 Department of Clinical and Experimental Sciences, ASST Spedali Civili di Brescia, University of Brescia, 25123 Brescia, Italy; 3 Radiation Oncology Department, ASST Spedali Civili di Brescia, University of Brescia, 25123 Brescia, Italy; 4 Department of Physiology and Pharmacology, Karolinska Institutet, 17177 Stockholm, Sweden; 5 Precision Medicine Research Center, School of Pharmacy, Binzhou Medical University, Laishan District, Guanhai Road 346, Yantai 264003, China; 6 Department of Hematology and Oncology, Cell and Gene Therapy, Bambino Gesù Children’s Hospital, IRCCS, 00165 Rome, Italy; 7 Department of Radiotherapy, Policlinico Umberto I, “Sapienza” University of Rome, 00161 Rome, Italy

**Keywords:** Akt, DNA repair, 2-deoxy-D-glucose, lovastatin, rhabdomyosarcoma

## Abstract

In pediatric rhabdomyosarcoma (RMS), elevated Akt signaling is associated with increased malignancy. Here, we report that expression of a constitutively active, myristoylated form of Akt1 (myrAkt1) in human RMS RD cells led to hyperactivation of the mammalian target of rapamycin (mTOR)/70-kDa ribosomal protein S6 kinase (p70S6K) pathway, resulting in the loss of both MyoD and myogenic capacity, and an increase of Ki67 expression due to high cell mitosis. MyrAkt1 signaling increased migratory and invasive cell traits, as detected by wound healing, zymography, and xenograft zebrafish assays, and promoted repair of DNA damage after radiotherapy and doxorubicin treatments, as revealed by nuclear detection of phosphorylated H2A histone family member X (γH2AX) through activation of DNA-dependent protein kinase (DNA-PK). Treatment with synthetic inhibitors of phosphatidylinositol-3-kinase (PI3K) and Akt was sufficient to completely revert the aggressive cell phenotype, while the mTOR inhibitor rapamycin failed to block cell dissemination. Furthermore, we found that pronounced Akt1 signaling increased the susceptibility to cell apoptosis after treatments with 2-deoxy-D-glucose (2-DG) and lovastatin, enzymatic inhibitors of hexokinase, and 3-hydroxy-3-methyl-glutaryl-coenzyme A reductase (HMGCR), especially in combination with radiotherapy and doxorubicin. In conclusion, these data suggest that restriction of glucose metabolism and the mevalonate pathway, in combination with standard therapy, may increase therapy success in RMS tumors characterized by a dysregulated Akt signaling.

## 1. Introduction

The signaling network defined by the serine/threonine kinase Akt forms a complex circuitry involved in physiological and pathological settings in nearly every organ system [1]. Upstream Akt phosphorylation is mediated by PI3K or phosphoinositide-dependent kinases (PDK), which, in turn, are activated by growth factors, nutrient availability, inflammation, and DNA damage. In turn, Akt signals through downstream effectors such as mTOR, glycogen synthase kinase 3 beta (GSK3β), or forkhead box protein O1 (FOXO1) [1]. In cancer, abnormally activated Akt/mTOR signaling is of paramount importance [2,3], allowing tumor cells to grow independently of exogenous growth stimuli, maximizing nutrient uptake and influencing metabolic plasticity [4,5] and contributing to multidrug resistance mechanisms [6]. RMS is a solid tumor affecting childhood and accounts for approximately 5% of all pediatric cancers [7]. It is thought to arise primarily from progenitor cells of mesenchymal and skeletal muscle lineages that carry genetic lesions, and it is typically characterized by expression of myogenic transcription factors, such as MyoD, Myf-5, and myogenin, which fail to promote correct differentiation [8,9]. The two most common subtypes are called embryonal (ERMS) and alveolar (ARMS), the latter characterized by PAX3-FOXO1 and PAX7-FOXO1 gene fusions resulting from chromosomal translocations and with a worse prognosis [10,11,12]. ERMS tumors are genetically more heterogenous and lack pathognomonic fusion genes. Most ERMS contain activating mutations for receptor tyrosine kinase, PI3K, and RAS, in addition to loss of function mutations in P53 [13,14,15,16]. RMS therapy requires a multimodal approach, consisting of surgery, radiotherapy, and chemotherapy (vincristine, dactinomycin, and cyclophosphamide/ifosfamide), resulting in an overall 5-year survival of >70% for localized RMS tumors, while metastatic disease has an unfavorable outcome. Mutations in the PI3K catalytic subunit [15,16], and Fibroblast Growth Factor Receptor 4 (FGFR4) [14,17], and loss of phosphatase and tensin homolog (PTEN) [18] are found recurrently in RMS to activate the PI3K/Akt/mTOR pathway, which is negatively associated with patient survival [19,20,21,22]. The Akt through mTOR complex regulates several important processes, including protein synthesis and inhibition of autophagy [23,24], and mechanisms of cell migration, invasion, and metastasis [25,26,27]. Since promising results were achieved by targeting of the Akt pathway in RMS [28,29,30,31,32,33], increasing the effectiveness of treatments aimed at blocking this signaling network represents the challenge for the treatment of advanced and metastatic RMS disease [34,35,36]. In this study, by means of in vitro and in vivo xenograft assays, we provide evidence that hyperactive Akt1 signaling in the human ERMS RD line increases cell proliferation, metastatic dissemination, and DNA repair mechanisms. Furthermore, we also demonstrated that oncogenic Akt1 signaling results in marked cell vulnerability to 2-DG and lovastatin, suggesting that glucose and mevalonate pathway metabolites are required for Akt1-driven RMS aggressiveness.

## 2. Materials and Methods

### 2.1. Reagents

The compounds LY294002, PD98059, rapamycin, 2-DG, and lovastatin were from Sigma Aldrich (Milan, Italy). MK2206 was from Aurogene (Rome, Italy) and doxorubicin from Lifetechnologies (Milan, Italy). Antibodies to pAkt-S473 (MAB-94111), Akt (MAB-94320), pp70S6K-Thr389 (AB-83589), pGSK3β-Ser9 (MAB-94145), and pDNA-PK-S2056 (ABP-0621) were from Immunological Sciences (Rome, Italy). Antibodies to pErk1/2-Tyr204 (sc-7383), Erk1/2 (sc-135900), MyoD (sc-377460), and MHC (sc-32732) were from Santa Cruz Biotechnology (Dallas, TX, USA). Anti-Ki67 (#12-5699-42) was from Invitrogen (Milan, Italy). Anti-pH2AX-Ser139 (9718) was from Cell Signaling Technology (Danvers, MA, USA). Anti-α-Tubulin (T5168) was from Sigma Aldrich. Secondary antibodies, anti-mouse (sc-516102) and anti-rabbit (sc-2357), were from Santa Cruz Biotechnology (Dallas, TX, USA). All the standard reagents were from Sigma Aldrich (Milan, Italy), unless otherwise stated.

### 2.2. Plasmids 

The pBabe-puromycin plasmid bearing a human myrAkt1 gene insert was sequenced by the Illumina technology platform (Illumina, San Diego, CA, USA). The same plasmid without insert was used as empty control vector.

### 2.3. Cell Cultures 

Human RD cells, purchased at the European Collection of Cell Cultures (ECACC), were cultured in high-glucose Dulbecco’s Modified Eagle’s Medium (DMEM) supplemented with 100 mg/mL penicillin/streptomycin and 10% fetal bovine serum (FBS) (Lifetechnologies, Milan, Italy), using standard conditions (humified incubator at 37 °C with 5% CO_2_). To induce myogenic differentiation, 80% of confluent cells were switched to a DMEM supplemented with 2% horse serum (HS) (Lifetechnologies, Milan, Italy), which was renewed daily. RD cells were stably transfected using Lipofectamine 3000 reagent (Lifetechnologies, Milan, Italy), according to the manufacturers’ instructions. The generated myrAkt1 clones showed similar cell behavior.

### 2.4. Immunoblotting

Whole-cell lysates were prepared using a RIPA lysis buffer (20 mM Tris-HCl at pH 7.6, 1% Nonidet P40, 0.5% sodium deoxycholate, 0.1% SDS, 50 mM NaCl) added with phosphatase inhibitors (1 mM Na_3_VO_4_ and 4 mM NaF), and a cocktail of protease inhibitors (Roche, Milan, Italy). Lysates were sonicated, clarified by centrifugation (12,000× *g* for 10 min at 4 °C) and protein concentration was determined using a Bradford assay. Equal amounts of protein samples were boiled at 99 °C for 5 min before SDS-PAGE, followed by transfer to polyvinylidene fluoride (PVDF) membranes (Life Technologies, Milan, Italy). Then, the membranes were blocked with TBS with 0.1% Tween-20 (TBS-T) and 5% milk for 15 min at room temperature (RT) and incubated with the primary antibody (overnight at 4 °C). Following TBS-T washes, the membranes were incubated with HRP-conjugated secondary antibody (for 1 h at RT) and then TBS-T washed again. Proteins were detected using enhanced chemiluminescence (ECL) (GeneSpin, Milan, Italy). Band densitometry was calculated using the Gel Pro Analyzer 4 software (MediaCybernetics Inc., Rockville, MD, USA).

### 2.5. Immunofluorescence Analysis

Cells were cultured onto 12 mm glass coverslips. Samples were then fixed with paraformaldehyde (PFA) solution (3% PFA in PBS) (for 20 min at 4 °C), permeabilized with Triton X-100 (0.1% Triton X-100 in PBS) (for 10 min at RT) and blocked with bovine serum albumin (BSA) solution (1% BSA in PBS with 0.1% sodium azide) (for 30 min at RT) prior to incubation with primary antibody (for 3 h at RT protected from light). Following BSA-washes, cells were incubated with secondary antibody (for 45 min at RT protected from light). Nuclei were counterstained with Hoechst dye (for 30 s at RT) and samples were mounted on slides using Mowiol mounting media. Images were acquired by a fluorescence Axiovert microscope (Carl Zeiss, Oberkochen, Germany) using the ImagePro Plus software (Media Cybernetics, Inc. Rockville, MD, USA). 

### 2.6. Crystal Violet Assay

Cells, seeded into 24-well plates in triplicates, were fixed with PFA solution (for 20 min at 4 °C) and stained with crystal violet solution (0.2% crystal violet in PBS with 20% methanol) (for 10 min at RT). Cells were washed with deionized water and solubilized in SDS solution (1% SDS in PBS). Plates were shaken until complete dissolution was achieved and then absorbance was measured by reading the plate at a 595 nm emission wavelength. 

### 2.7. Clonogenic Assay

After exposure of growing cells to ionizing radiation treatment using an X-ray linear accelerator (dose rate of 2 Gy/minute), cells were detached with trypsin and reseeded into 6-well plates (1 × 10^3^) in triplicate. Plates were incubated for 10 days under standard conditions. Grown colonies were fixed with PFA solution (for 20 min at 4 °C) and then stained with crystal violet solution (for 10 min at RT). Pictures were taken after staining with the dye and then the samples were solubilized using an SDS solution. 

### 2.8. Zymography Assay

Cells were seeded into 60 mm dishes (2 × 10^5^) 24 h prior to incubation with serum deprived DMEM. After 24 h, conditioned media were collected and analyzed by SDS-PAGE in 0.1% gelatin-8% acrylamide gels under non-reducing conditions. After electrophoresis, gels were washed with Triton X-100 solution (2.5% Triton X-100 in PBS) (for 2 h at RT) and incubated with Collagenase Buffer (50 mM Tris-HCl, 5 mM CaCl_2_, 0.02% sodium azide, 0.005% Brij 35, 1 μM ZnCl_2_) (for 24 h at 37 °C). Gels were stained with Coomassie blue solution (0.1% Coomassie blue in deionized water with 40% ethanol and 10% acetic glacial acid) (for 1 h at RT) and then incubated with destaining solution (30% methanol in deionized water with 10% acetic glacial acid) until MMP-2 and MMP-9 bands were detected.

### 2.9. Wound Healing Assay

Cells were seeded into 6-well plates (2 × 10^5^) in triplicates. After 24 h, confluent cells were scratched with a sterile 200 µL micropipette tip. Images of wound healing were taken at different time points using an inverted light microscope (Olympus IX50; Olympus, Tokyo, Japan) with cellSens Software (Olympus, Tokyo, Japan). The wounding area was quantified by using ImageJ software. 

### 2.10. Neutral Red Assay

Cells were seeded in 96-well plates in triplicates (1.5 × 10^3^) prior to treatment with doxorubicin, 2-DG, and lovastatin after 24 h. After a 48-h long treatment, media were replaced with DMEM added with 5% FBS and 40 μg/mL neutral red dye for 2 h. Then, cells were PBS-washed and incubated with a destaining solution (50% ethanol in deionized water with 1% acetic glacial acid). Plates were shaken until complete dye extraction was achieved and then absorbance was measured by reading the plate at 540 nm emission wavelengths. 

### 2.11. Flow Cytometric Analyses

Cell apoptosis was assessed using an Annexin V/Propidium Iodide (PI) apoptosis-detection kit (Immunostep Biotec, Salamanca, Spain), according to the manufacturer’s instructions. Cells were seeded into 6-well plates in duplicate and processed as indicated. After 48 h of treatment, cells were collected into flow cytometry tubes, PBS washed, resuspended in Binding Buffer and double stained with Annexin-V-FITC/PI. Cytofluorimetric analysis was performed using a MACSQuant Analyzer. Cell debris, doublets, and aggregates were excluded from analysis, and 20,000 events per sample were analyzed. To evaluate Ki67 expression, cells were permeabilized with 0.1% saponin in PBS (for 10 min at 4 °C). Then, cells were washed, incubated with anti-human Ki67-PE antibody (for 30 min at RT), and analyzed with the MACSQuant Analyzer.

### 2.12. Zebrafish Xenograft assay

The transgenic zebrafish *kdr1:EGFP* line [37] was maintained according to international protocols (http://ZFIN.org; accessed on 1 January 2022) and national guidelines, following protocols approved by the local Committee OPBA and authorized by the Ministry of Health (298/2018). Zebrafish were maintained at 28 °C under a 14 h light/10 h dark cycle with a pH value of 7.0–7.5 and conductivity of between 400–500 µs. Fish were fed with a combination of granular food (Special Diet Services, Witham, UK) and freshly prepared Artemia sp. (Special Diet Services, Witham, UK). Embryos were treated with 0.003% 1-phenyl-2-thiourea (PTU) to prevent pigmentation. Zebrafish embryos at 2 days post fertilization (dpf) were anaesthetized with 200 mg/L tricaine and microinjected with CM-DiI-labeled tumor cells (~240 cells in 4 nL of PBS per embryo) into the subperidermal space of the yolk sac using the electronic microinjector FemtoJet coupled with the InjectMan N12 manipulator (Eppendorf Italia, Milan, Italy). After cell injection, embryos were maintained at 33 °C. To test the efficacy of synthetic compounds on xenografted tumors, embryos at 1 day post injection (dpi) were kept in water added with optimal drug doses, as determined after performing the Fish Embryo Toxicity test. Pictures of embryos were acquired using an AXIO Zoom V16 fluorescence stereomicroscope (Carl Zeiss, Germany) equipped with a PlanNeoFluar Z 1x/o.25 FWD 56 mm lens and Zen Pro Software. Quantification of the tumor area was performed by using Image J Fiji (https://imagej.net/software/fiji; accessed on 1 January 2022). The embryos were then fixed with PFA solution (PFA 4% with glutaraldehyde 2%) and processed for confocal analysis by using a confocal microscope with photo-multiplier tube detectors (LSM 510 Meta; Carl Zeiss, Germany) using the LSM510 Meta software (Carl Zeiss, Germany).

### 2.13. Statistical Analysis

All error bars represent standard deviation. For pairwise comparisons, a two-tailed Student’s *t* test was used, whereas a one-way Anova test was used to compare the means among three or more groups using GraphPad Prism 5 software (GraphPad Software, San Diego, CA, USA). The significance threshold was a *p*-value < 0.05. 

## 3. Results

### 3.1. Hyperactive Akt1 Signaling Suppresses Myogenic Differentiation and Enhances Growth Rate and Clonogenicity in RD Line

**Figure 1:**
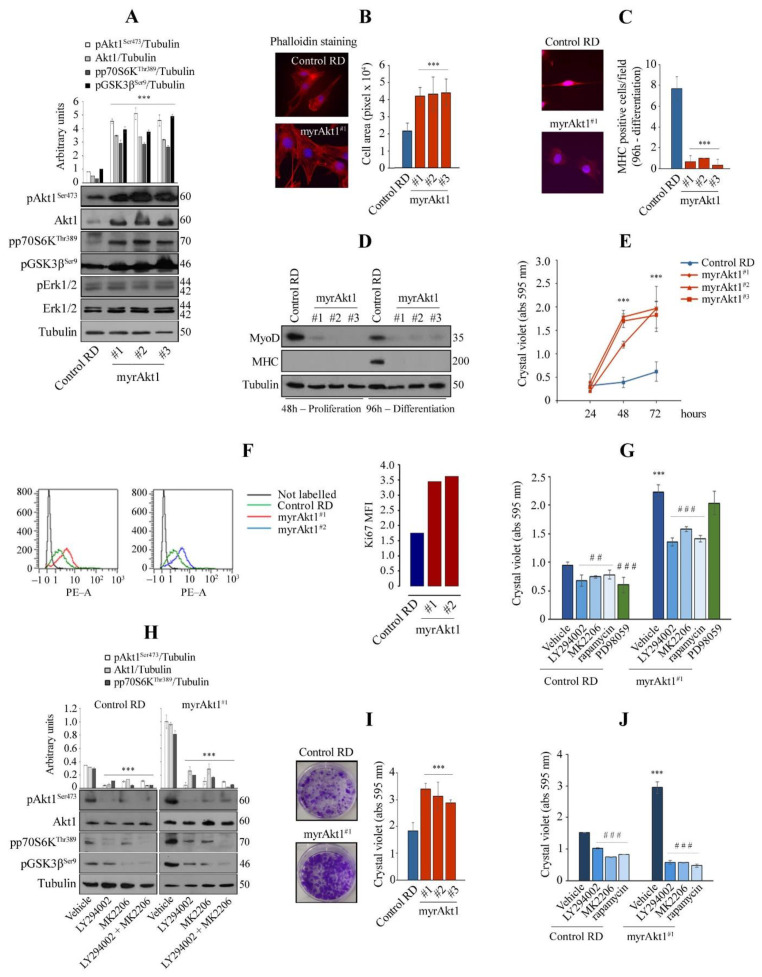
Analysis of cell differentiation and growth in the RD line expressing a myrAkt1 form. (**A**) Control and myrAkt1 cells (1.5 × 10^5^), seeded into 60 mm dishes, were left to proliferate for 48 h before collecting protein samples. Activation of Akt1 signaling was determined by IB with the indicated antibodies (*n* = 2). Data are mean ± SEM, *** *p*-value < 0.0001; one-way Anova test. (**B**) Under the same conditions seen above, cells were seeded on coverslips and stained with fluorescent red phalloidin. Representative images were taken using a fluorescent microscope at 63× magnification (*n* = 2). Quantification of the cell area was calculated by using ImagePro Plus software. Data are mean ± SEM, *** *p*-value < 0.0001; one-way Anova test. (**C**) Control and myrAkt1 cells (2 × 10^5^), seeded onto coverslips in 60 mm dishes, were left to proliferate until reaching confluence (48 h), before treatment with DM (up to 96 h). Then, MHC staining was analyzed by IF. Reported quantification is relative to the average number of MHC-positive cells resulting from at least 10 different fields (*n* = 2). Data are mean ± SEM, *** *p*-value < 0.0001; unpaired Student’s *t*-test. (**D**) IB for detection of MyoD and MHC was performed on proliferating and differentiating cells at the indicated times, respectively (*n* = 2). (**E**) After seeding the cells (1.5 × 10^4^) in 24-multiwell plates, the cell growth was evaluated over a time-course by crystal violet incorporation (*n* = 3). Data are mean ± SEM, *** *p*-value < 0.0001; one-way Anova test. (**F**) Control and myrAkt1 cells (1 × 10^5^) were seeded into 100 mm dishes and left to proliferate for 24 h in GM before starvation with a GM supplemented with 5% FBS. Ki67 quantitative fluorescence was measured in sorted cells after 72 h by FACS analysis (*n* = 2). (**G**) Control and myrAkt1 cells (1.5 × 10^4^), seeded in 24-multiwell plates, were left to proliferate in GM for 24 h before acute treatment with 10 µM LY294002, 10 µM MK2206, 100 nM rapamycin, 10 µM PD98059, or DMSO vehicle. After 72 h of cell growth, crystal violet incorporation was quantified (*n* = 3). Data are mean ± SEM, *** *p*-value < 0.0001; unpaired Student’s *t*-test vs. control RD line. ## *p*-value < 0.001; ### *p*-value < 0.0001; one-way Anova test vs. DMSO-treated cells. (**H**) Control and myrAkt1 cells (1.5 × 10^5^)**,** seeded into 60 mm dishes, were maintained for 24 h in GM before acute treatment with 10 µM LY294002, 10 µM MK2206, a combination of 5 µM LY294002 and 5 µM MK2206 or a DMSO vehicle. Activation of Akt1 signaling was determined by IB with the indicated antibodies after 72 h (*n* = 2). Data are mean ± SEM, *** *p*-value < 0.0001; one-way Anova test. (**I**) Pictures showing the clonogenic capacity of control and myrAkt1 cells. Single colonies were left to grow for 10 days in 6-multiwell plates prior to crystal violet incorporation (for details, see Material and Methods). The graph (right panel) shows the crystal violet quantification (*n* = 3). Data are mean ± SEM, *** *p*-value < 0.0001; one-way Anova test. (**J**) Cell growth was evaluated by clonogenic assay after 2-h pretreatment with 10 µM LY294002, 10 µM MK2206, 100 nM rapamycin, or a DMSO vehicle. Quantification was performed by crystal violet incorporation (*n* = 2). Data are mean ± SEM, *** *p*-value < 0.0001; unpaired Student’s *t*-test *vs* control RD line. ### *p*-value < 0.0001; one-way Anova test vs. DMSO-treated cells.

Since Akt1 is the preferentially expressed isoform in the RD cell line [38], we transfected a vector encoding a myrAkt1 form to mimic activated Akt signaling. As shown by immunoblotting (IB), the selected clones (referred to as hashtag-numbered myrAkt1) cultured in growth medium (GM) showed an approximately four to five-fold increase in total Akt1 and Ser^473^-phosphorylated form compared to control cells transfected with an empty vector (Figure 1A), confirming that myrAkt1 acts as a constitutively activated form. Consequently, myrAkt1 cells also showed increased phosphorylation of GSK3β and p70S6K, two downstream targets of Akt1 signaling (Figure 1A). Although RD cells carry NRAS mutations, activating the extracellular regulated kinase (ERK) pathway [39], myrAkt1 expression did not change the levels of phosphorylated ERK1/2 compared to control (Figure 1A). By analysis of cell morphology with phalloidin staining, the results showed that the mean cell size in the myrAkt1 clones was approximately doubled compared to control cells, as detected by fluorescent microscopy (Figure 1B). Next, to assess whether Akt1 signaling could affect myogenic capacity, the cells were cultured in a differentiation medium (DM). After 4 days, the myrAkt1 cells showed no morphological signs of differentiation compared to control cells that instead appeared to be partially elongated (not shown). This was confirmed by the IF and IB analysis, which showed the lack of myosin heavy chain (MHC) in myrAkt1 cells compared to control (Figure 1C,D). Furthermore, myrAkt1 cells showed a MyoD deficiency during both proliferation and differentiation conditions, compared to control (Figure 1D). By crystal violet assay, the growth rate of the myrAkt1 cells was markedly pronounced compared to control, as detected over a 72-h time course (Figure 1E). Sustained growth capacity was confirmed by a single-cell assay after Ki67 staining. All sorted myrAkt1 cells showed higher Ki67 expression, which was found to be approximately double that of control cells (Figure 1F). Acute treatments with the PI3K inhibitor LY294002, the Akt competitive inhibitor MK2206, and the mTOR inhibitor rapamycin significantly slowed the proliferation rate of myrAkt1 and control cells (Figure 1G). In contrast, treatment with PD98059 (an upstream ERK1/2 inhibitor) did not produce any effect on the cell growth of myrAkt1 cells, while it slowed the proliferation of control cells (Figure 1G), suggesting that increased Akt1 activity stimulates cell growth regardless of the ERK pathway. By IB, we tested the efficacy of acute inhibitory treatments on some downstream targets of Akt1. Single or combined treatments with LY294002 and MK2206 resulted in a significant and similar reduction of phosphorylated Akt1, p70S6K, and GSK3β kinases, in both control and myrAkt1 cells (Figure 1H). Therefore, in all subsequent experiments, they were administered as single treatments. Finally, by means of the colony formation assay, we observed an increase in the clonogenicity of myrAkt1 cells compared to control cells (Figure 1I), which was inhibited by treatments with LY294002, MK2206, or rapamycin (Figure 1J). Taken together, these results suggest that myrAkt1 expression in RD cells is detrimental to myogenic differentiation, while increasing cell growth and clonogenicity via the Akt1/mTOR pathway.

### 3.2. MyrAkt1 Enhances Tumor Cell Dissemination In Vitro and In Vivo

**Figure 2:**
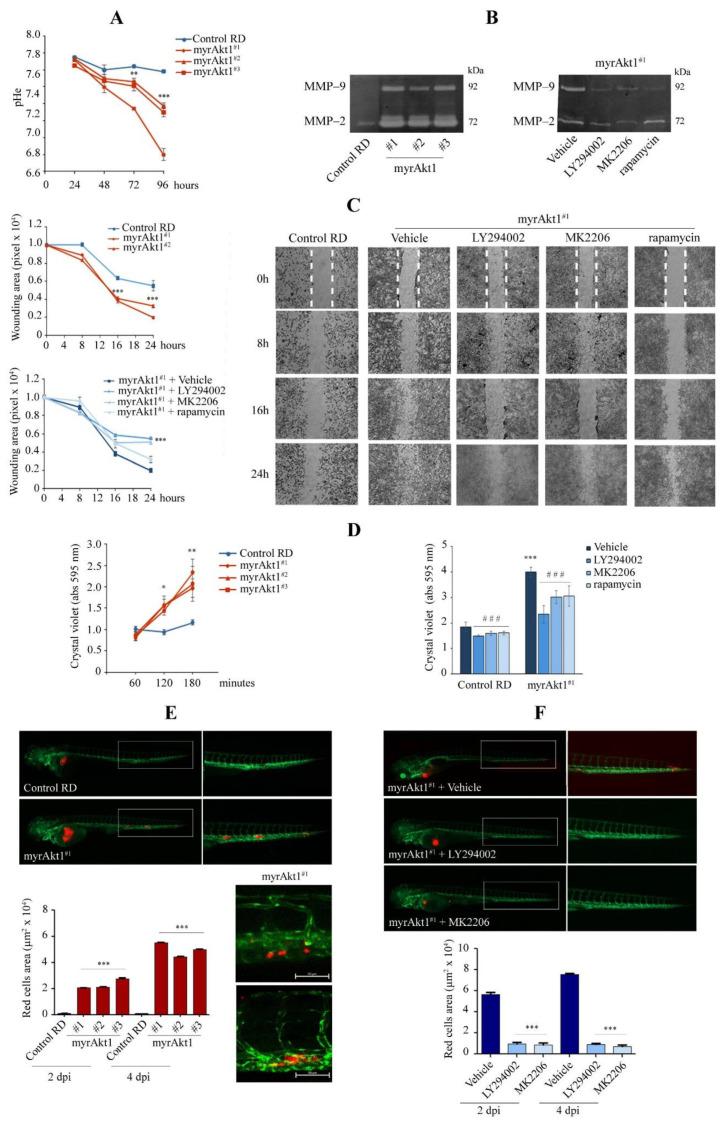
Analysis of cell invasion and migration by in vitro and in vivo assays. (**A**) Control and myrAkt1 cells (6 × 10^4^) were seeded into 6-multiwell plates and left to proliferate up to 96 h. At the indicated time points, the medium pH was measured (*n* = 2). Data are mean ± SEM, ** *p*-value < 0.001; *** *p*-value < 0.0001; one-way Anova test. (**B**) Gel zymogram depicting differences in MMP-2 and -9 content between control and myrAkt1 cells (left panel) (*n* = 2). MMP-2 and -9 expression was also assessed in myrAkt1 cells pre-treated with 10 µM LY294002, 10 µM MK2206, or 100 nM rapamycin for 24 h (right panel) (*n* = 2). (**C**) The migration capacity of control and myrAkt1 cells was evaluated by wound healing assay over a time course of 24 h (top graph). The increased migratory cell behavior of myrAkt1 cells was evaluated after 2 h-pretreatment with 10 µM LY294002, 10 µM MK2206, 100 nM rapamycin, or DMSO vehicle (bottom graph) (*n* = 3). Representative pictures showing the migration front were taken at 10x magnification. The edges of the wound at time 0 h are identified as dotted white lines. Data are mean ± SEM, *** *p*-value < 0.0001; one-way Anova test. (**D**) After seeding control and myrAkt1 cells in 24-multiwell plates (3 × 10^4^), cell adhesion was evaluated by crystal violet in the absence or presence of 2 h pre-treatment with 10 µM LY294002, 10 µM MK2206, 100 nM rapamycin, or a DMSO vehicle (left and right graphs, respectively) (*n* = 2). Data are mean ± SEM, * *p*-value < 0.05; ** *p*-value < 0.001; *** *p*-value < 0.0001; unpaired Student’s *t*-test vs. control RD line. ### *p*-value < 0.0001; one-way Anova test vs. DMSO-treated cells. (**E**) CM-Dil fluorescent labeled control and myrAkt1 cells (~250) were engrafted into the yolk sac of zebrafish embryos. Representative images of cell dissemination were taken after 4 dpi using a fluorescent Axio Zoom V16 microscope at 20× and 32× magnification. Quantification of migrated tumor cells was calculated after 2 and 4 dpi by using Noldus DanioScope TM software (*n* = 3). Data are mean ± SEM, *** *p*-value < 0.0001; one-way Anova test. (**F**) Fluorescent labeled myrAkt1 cells were pretreated with 10 µM LY294002, 10 µM MK2206 or DMSO vehicle 24 h prior to yolk injection into zebrafish embryos. Xenografted embryos were maintained in water added with 2.5 µM LY294002, 5 µM MK2206, or DMSO vehicle until 4 dpi. Representative images were taken after 4 dpi at 20× and 32× magnification. Quantification of migrated tumor cells was calculated after 2 and 4 dpi (*n* = 2). Data are mean ± SEM, *** *p*-value < 0.0001; one-way Anova test.

During cell growth up to 96 h, we observed that the extracellular pH of myrAkt1 cells reached markedly acidic values, between 6.8 and 7.4, compared to the mean value of 7.8 detected in the control cells (Figure 2A). Since pH acidification is recognized as a feature of increased tumor dissemination [40], we performed cell invasion, migration, and adhesion assays. In myrAkt1 cells, the metalloproteases MMP-2 and MMP-9 were significantly increased compared to control cells (Figure 2B), and their expression was reduced by LY294002 or MK2206 treatment, as detected by the zymography assay (Figure 2B). In contrast, rapamycin-treated myrAkt1 cells showed a slight decrease in MMP-9 expression only (Figure 2B). We then evaluated cell migration by a wound healing assay. After scratching the cell monolayer, repair of the injured area was faster and more efficient (75–93% of total area) in the myrAkt1 cells compared to control (32%), as detected over a 24-h time-course (Figure 2C). Furthermore, pretreatment of myrAkt1 cells with LY294002 or MK2206 abrogated the repair capacity, while rapamycin had a minor effect (Figure 2C). Finally, the increased cell adhesion detected in the myrAkt1 cells was abrogated by LY294002, MK2206, and rapamycin (Figure 2D). To evaluate metastatic spread in vivo, cells were labeled with the fluorescent CM-DiI [41] and grafted into the yolk sac of zebrafish embryos at 2 dpf. Following myrAkt1 cell injection, we observed the formation of metastatic foci spreading along the zebrafish body, as visualized at 2 and 4 dpi by fluorescent microscopy (Figure 2E). Tumor dissemination of myrAkt1 cells was significantly decreased by 24-h pretreatments with LY294002 or MK2206 prior to transplantation (Figure 2F). It should be noted that the grafted embryos were kept in water supplemented with 2.5 µM LY294002 and 5 µM MK2206 for 4 days without reporting any side effects. Conversely, we were unable to assess the effects of rapamycin, given the edema observed in all treated embryos (not shown).

### 3.3. The Akt1/mTOR/DNA-PK Signaling Axis Correlates with Increase in DNA Repair

**Figure 3:**
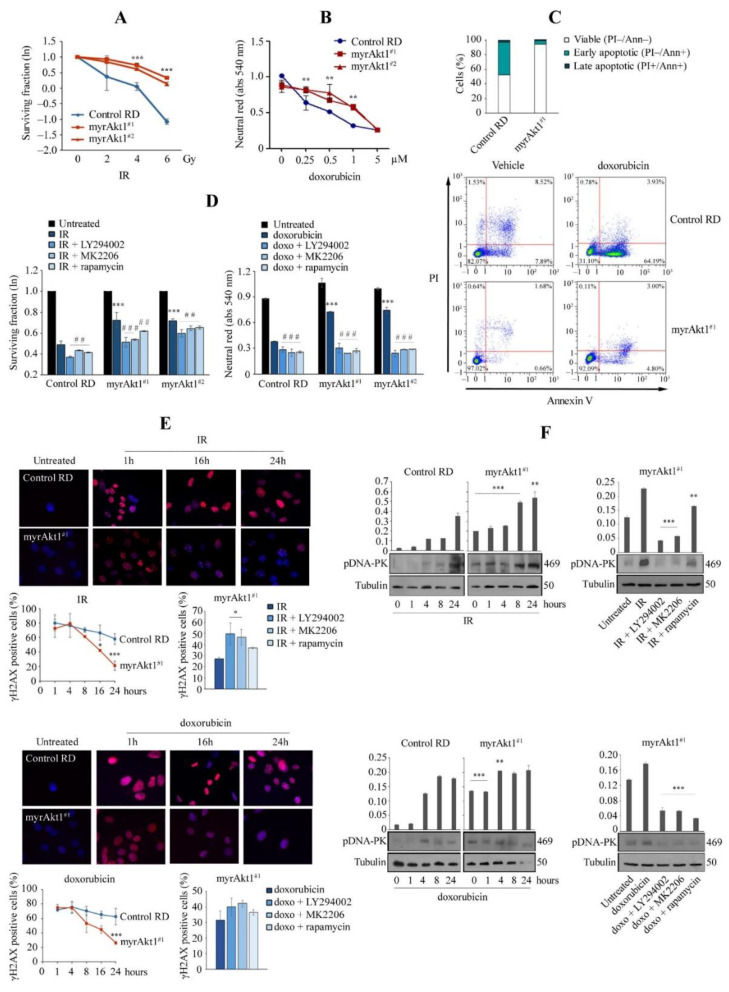
Analysis of apoptosis and DNA damage mechanisms in response to genotoxic stress agents. (**A**) Control and myrAkt1 cells (2 × 10^5^), seeded into 60 mm dishes, were left 24 h in GM and then exposed to increasing IR doses. Quantification of colony formation by a crystal violet assay was expressed as a natural logarithm, setting control to 1 (*n* = 3). Data are mean ± SEM, *** *p*-value < 0.0001; one-way Anova test. (**B**) Neutral red assay was performed to measure cell viability in control and myrAkt1 clones treated for 48 h with increasing doxorubicin doses (*n* = 3). Data are mean ± SEM, ** *p*-value < 0.001; one-way Anova test. (**C**) Cells (1 × 10^5^) were seeded into 6-multiwell plates. After 24 h, cells were treated with 1 µM doxorubicin or DMSO vehicle. After 48 h, cells were stained with PI and Annexin V. The percentages of viable, early, and late apoptotic cells were calculated by FACS analysis and are reported in the graph (*n* = 2). (**D**) Clonogenic and neutral red assays (left and right graphs, respectively) were performed to measure viability of cells preincubated for 2 h with 10 µM LY294002, 10 µM MK2206, 100 nM rapamycin, or the DMSO vehicle before irradiation (4 Gy) and doxorubicin treatment (1 µM) (*n* = 3). Data are mean ± SEM, *** *p*-value < 0.0001; unpaired Student’s *t*-test vs. control RD cells. ## *p*-value < 0.001; ### *p*-value < 0.0001; one-way Anova test vs. DMSO-treated cells. (**E**) Control and myrAkt1 cells (1.5 × 10^5^), seeded onto coverslips in 60 mm dishes, were left to proliferate in GM for 48 h before irradiation (4 Gy) or treatment with doxorubicin (1 µM). Nuclear γH2AX staining was evaluated by IF analysis over a time course of 24 h. Representative images were taken at 63× magnification. The reported quantification in the bottom left graphs is relative to the average number of γH2AX-positive cells counted in 10 different fields (*n* = 2). As reported in the bottom right graphs, nuclear γH2AX staining was evaluated in cells pre-treated with 10 μM LY294002, 10 μM MK2206, 100 nM rapamycin, or the DMSO vehicle for 2 h before irradiation (4 Gy) or administration of doxorubicin (1 µM) (*n* = 2). Data are mean ± SEM, * *p*-value < 0.05; *** *p*-value < 0.0001; unpaired Student’s *t*-test. (**F**) Control and myrAkt1 cells (1.5 × 10^5^), seeded into 60 mm dishes, were left to proliferate in GM for 48 h before irradiation (4 Gy) (top panel) or treatment with doxorubicin (1 µM) (bottom panel). At the indicated time points, cells were harvested, and protein homogenates were blotted to perform IB for pDNA-PK (*n* = 2). Under the same conditions seen above, cells were pre-treated with 10 μM LY294002, 10 μM MK2206, 100 nM rapamycin, or the DMSO vehicle for 2 h prior to irradiation (4 Gy) (left panel) or doxorubicin administration (1 µM) (right panel). IB was performed using protein homogenates from cells harvested after 8 h (*n* = 2). Data are mean ± SEM, ** *p*-value < 0.001; *** *p*-value < 0.0001; unpaired Student’s *t*-test.

We evaluated the effects of radiotherapy and the anthracycline agent doxorubicin [42], inducers of DNA double-strand breaks (DSBs), on cell viability. After radiotherapy with doses ranging from 2 to 6 Gy, cell survival was greater in myrAkt1 cells than in control, as detected by the clonogenic assay (Figure 3A). The D50 values (dose required to reduce survival to 50%) were 5 and 2.6 Gy for myrAkt1 and control cells, respectively. Similarly, after doxorubicin treatment with doses ranging from 0.25 to 5 µM, cell viability was higher in myrAkt1 cells as measured by the neutral red assay (Figure 3B). In this case, the D50 values were 3 and 1 µM for myrAkt1 and control cells, respectively. FACS analysis on doxorubicin-treated cells revealed a higher percentage of viable cells in the myrAkt1 line (up to 90%) compared to the control (50%), which showed early apoptotic signs (Figure 3C). Furthermore, in the vehicle-treated cells, the percentage of cells with basal apoptotic signs was approximately 2.3% in myrAkt1 and 16.3% in the control (Figure 3C). In myrAkt1 cells, sensitivity to IR and doxorubicin was significantly restored by acute 2-h pre-treatments with LY294002, MK2206 or rapamycin, as shown by the clonogenic and neutral red assays, respectively (Figure 3D). These data clearly supported a protective role of Akt1 signaling, and therefore we sought to assess whether the increased resistance of myrAkt1 cells to genotoxic stress was due to reduced DNA damage rather than increased DNA repair capacity. For DNA damage quantification, we performed IF analysis of the nuclear γH2AX, which binds to DSBs following genotoxic stress [43]. The results showed that nuclear γH2AX was detectable in a similar manner in both control and myrAkt1 cells already after short-time exposure (1 h) to IR or doxorubicin (Figure 3E). However, the γH2AX nuclear staining disappeared faster in the myrAkt1 cells than in control cells, as visualized by IF images and quantification of nuclear γH2AX staining (Figure 3E, top pictures and bottom left graph). Furthermore, treatment with LY294002, MK2206, or rapamycin abrogated DNA repair in myrAkt1 cells, as shown by γH2AX nuclear staining (Figure 3E, bottom right graph), suggesting that Akt1/mTOR signaling increases the rate of DNA repair. Since Akt1 was shown to promote DNA repair primarily through the non-homologous end joining (NHEJ) pathway [44,45], we evaluated expression of DNA-PK. IB analysis showed that basal DNA-PK phosphorylation was already higher in untreated myrAkt1 cells compared to the control (Figure 3F). Furthermore, after treatment with IR or doxorubicin, DNA-PK phosphorylation increased and remained consistently higher in myrAkt1 cells over 24 h (Figure 3F). Again, treatment with inhibitors of the Akt1 pathway led to a decrease in DNA-PK phosphorylation (Figure 3F), confirming that Akt1/mTOR signaling contributes to the activation of the DNA repair machinery in RD cells.

### 3.4. Treatments with 2-DG and Lovastatin, by Triggering Apoptosis, Inhibit myrAkt1-Driven Tumorigenicity

**Figure 4:**
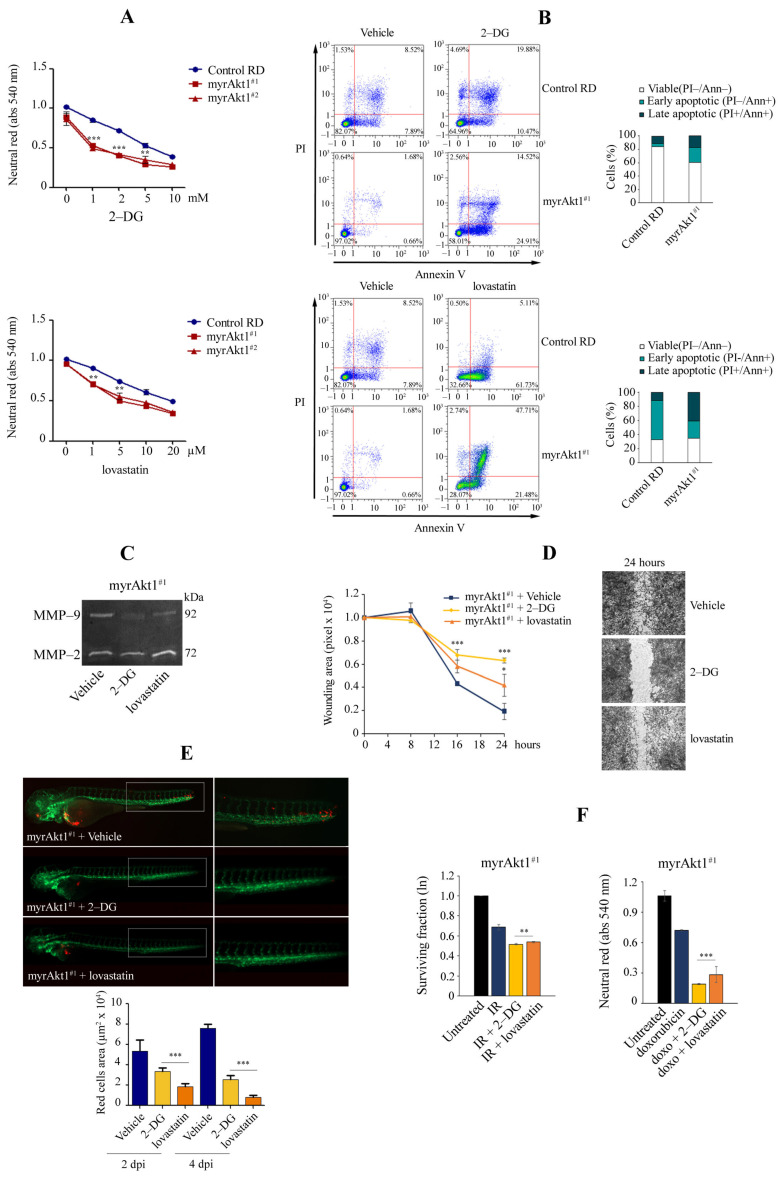
Effects of 2-DG and lovastatin on cell survival, cell dissemination, and radio- and chemotherapy. (**A**) Neutral red assay was performed to measure cell viability in control and myrAkt1 clones treated for 48 h with increasing 2-DG and lovastatin doses (*n* = 3). Data are mean ± SEM, ** *p*-value < 0.001; *** *p*-value < 0.0001; one-way Anova test. (**B**) Cells (1 × 10^5^) were seeded into 6-multiwell plates. After 24 h, cells were treated with 2 mM 2-DG and 10 µM lovastatin or DMSO vehicle. After 48 h, cells were stained with PI and Annexin V. The percentages of viable, early, and late apoptotic cells were calculated by FACS analysis and are reported in the graph (*n* = 2). (**C**) Gel zymogram depicting differences in MMP-2 and -9 expression in myrAkt1 cells treated with 2 mM 2-DG and 10 µM lovastatin or DMSO vehicle for 24 h (*n* = 2). (**D**) MyrAkt1 cells were pre-treated for 2 h with 2 mM 2-DG and 10 µM lovastatin or DMSO vehicle before wound induction. As depicted in the graph, the wound repair area was evaluated over a time-course of 24 h. Representative images were taken after 24 h (*n* = 2). Data are mean ± SEM, * *p*-value < 0.05; *** *p*-value < 0.0001; one-way Anova test. (**E**) CM-Dil fluorescent labeled myrAkt1 cells were pretreated with 2 mM 2-DG, 10 µM lovastatin or DMSO vehicle 24 h prior to yolk injection into zebrafish embryos. Xenografted embryos were maintained in water added with 50 µM 2-DG, 0.1 µM lovastatin, or the DMSO vehicle. Representative images were taken after 4 dpi at 20× and 32× magnification. Quantification of migrated tumor cells was calculated at 2 and 4 dpi (*n* = 3). Data are mean ± SEM, *** *p*-value < 0.0001; one-way Anova test. (**F**) Clonogenic and neutral red assays (left and right graphs, respectively) were performed to measure viability of cells preincubated for 2 h with 2 mM 2-DG, 10 µM lovastatin, or the DMSO vehicle before irradiation (4 Gy) and doxorubicin treatment (1 µM) (*n* = 3). Data are mean ± SEM, ** *p*-value < 0.001; *** *p*-value < 0.0001; one-way Anova test.

In cancer, elevated Akt1 signaling is thought to be responsible for intensified glycolysis [46] and the increase in the mevalonate pathway that causes high intracellular cholesterol production [47]. Therefore, we sought to assess whether treatments with 2-DG and lovastatin, inhibitors of hexokinase and HMGCR enzymes, could affect cell viability in myrAkt1 lines. Using the neutral red assay, we observed that both 2-DG (doses ranging from 1–10 mM) and lovastatin (doses ranging from 1–20 µM) significantly reduced cell viability in myrAkt1 lines compared to the control (Figure 4A). For myrAkt1 and control cells, the D50 values for 2-DG were 2 and 8 mM, and for lovastatin they were 10 and 25 µM, respectively. After 48 h of 2-DG treatment, FACS analysis revealed a higher percentage of apoptotic cells in the myrAkt1 cells (~40%), while only 29% of the control cells showed apoptotic signs (Figure 4B). After 48 h-treatment with lovastatin, more than 47% of myrAkt1 cells showed signs of late apoptosis compared with 5% in control cells (Figure 4B). We then evaluated the effects of 2-DG and lovastatin on cell invasion and migration. In myrAkt1 cells, both drugs resulted in a selective downregulation of MMP-9 expression (Figure 4C). The drugs also had a significant inhibitory effect on cell migration of myrAkt1 cells, with 2-DG showing the most pronounced inhibitory action, as detected by the wound healing assay (Figure 4D). These results were similarly observed in control cells (not shown). We therefore sought to evaluate whether pretreatment of myrAkt1 cells with 2-DG or lovastatin could influence the dissemination process in zebrafish embryos. As shown in representative images (Figure 4E), myrAkt1 cells pretreated with lovastatin and 2-DG failed to spread along the embryo bodies compared to the control cells. Also in this case, the grafted embryos were kept in water supplemented with 50 µM 2-DG and 0.1 µM lovastatin for 4 days without reporting any side effects. As reported by quantifying CM-DiI-labeled myrAkt1 cells after 2 and 4 dpi, we found that lovastatin has a greater effect than 2-DG on reducing the formation of distant metastatic foci (Figure 4E). Finally, drug pre-treatment significantly radio- and chemo-sensitized myrAkt1 cells to apoptotic cell death, as determined by the clonogenic and neutral red assays (Figure 4F). In particular, 2-DG appeared to be more effective than lovastatin. 

## 4. Discussion

Despite aggressive multimodal treatment, metastatic RMS has a poor prognosis, with an overall 5-year survival of less than 30% [35]. Unfavorable prognosis is often related to mutational activation of PI3K/Akt signaling [19,20,21], whose inhibition has in fact been shown to increase therapy sensitivity in a tumor subgroup, as revealed using patient-derived xenograft models [48]. However, understanding of the molecular mechanisms driven by Akt circuits is still limited, and current targeted therapies do not lead to complete tumor remission. The results shown in this work provide evidence that increasing the Akt pathway activation by myrAkt1 expression facilitates tumor growth, cell dissemination, and DNA repair in the ERMS RD line. At the same time, our data suggest that glucose availability and the mevalonate pathway are required to support myrAkt1-driven tumor aggressiveness. RMS is considered a tumor arising from mesenchymal cell precursors that fail to undergo proper skeletal muscle differentiation [9]. Akt signaling is required for cell cycle regulation [49] and plays a central role in skeletal muscle during myoblast proliferation, differentiation, and hypertrophy [50,51]. Akt1 isoform is considered a kinase favorable for early myogenic differentiation [52] and its dysregulation plays a role in muscle atrophy and diabetes type II-related sarcopenia [53,54]. Akt modulators are therefore expected to interfere with a variety of functions in the muscle. Our data show that pronounced Akt1 signaling in RD cells led to a high mitotic rate but limited the differentiation capacity, confirming recent findings from Granados et al. [55]. The identification of muscle-related differentiation is clinically relevant because some RMS cases lacking myogenic markers are virtually indistinguishable from the group of so-called undifferentiated soft-tissue sarcomas, which have a poor outcome compared with RMS [56]. In this regard, our experiments showed that mitotic myrAkt1 cells lost expression of MyoD, a myogenic transcription factor required for differentiation [57,58], suggesting that Akt1 signaling may increase tumorigenicity of RD cells by affecting MyoD expression or stability. In this context, silencing of MyoD was shown to increase cell death in RD cells [8], while myrAkt1 cells showed no signs of cell distress or cell mortality. This suggests that elevated Akt1 signaling could protect MyoD-deficient RD cells from apoptosis. Of note, it has recently been found that RD cell differentiation depends on coordinated activation of both the MKK6/p38 and Akt1 pathways [59], suggesting that Akt1 activation alone is not sufficient to elicit the myogenic program. By means of in vitro and in vivo assays, we demonstrated that myrAkt1 cells acquired increased cell growth, adhesion, and dissemination. Surprisingly, the mTOR inhibitor rapamycin had no significant effect on cell dissemination. In mammalian cells, mTOR functions as two physically and functionally distinct signaling complexes, mTOR complex 1 and 2 (mTORC1 and mTORC2), which differ in their protein components, cellular functions, and rapamycin sensitivity (reviewed in [60,61]). The rapamycin-sensitive mTORC1 has been shown to activate the p70S6K, in turn leading to increased cell growth and proliferation [23,24]. Rapamycin-insensitive mTORC2 instead mainly contributes to the regulation of the actin cytoskeleton essentially for cell migration and invasion [25,26,27]. In their recent work, Felkai et al. showed mTORC2 dominance in primary RMS samples by immunohistochemistry analysis [62], suggesting the need to test dual mTORC1/2 inhibitors for treatment of high grade, rapamycin-resistant RMS [63,64]. Our data, based on an inhibitory approach, suggest that mTORC1 signaling in RD cells may be required for cell growth and DNA damage repair, while mTORC2 for migration and invasion mechanisms. Previous works have indicated a role of Akt in the DNA repair mechanisms of RMS after radio- [65,66] and chemotherapy [67]. We hereby confirm the importance of the Akt1/mTOR axis in driving DNA repair through increased activation of the serine/threonine DNA-PK, a critical component of the non-homologous end joining (NHEJ) pathway [68]. Several inhibitors of DNA-PK have recently been developed [69,70], and using a dual inhibitor of mTOR kinase and DNA-PK called CC-115, currently being studied in phase I/II clinical trials, has been shown to radiosensitize melanoma cell lines [71]. In this regard, targeting the Akt1/mTOR/DNA-PK signaling axis could be critical for limiting ERMS tumor regrowth and recurrence. In dividing tumor cells, Akt activation is responsible for metabolic changes that increase energy production by aerobic glycolysis (Warburg effect) [46], while a part of glucose is used in the pentose phosphate pathway to produce ribose and NADPH dispensable for nucleotide and lipid synthesis and oxidative stress protection [72]. Our results showed that high Akt1 signaling conferred marked susceptibility to 2-DG and lovastatin, inhibitors of hexokinase and HMGCR that limit glycolysis and intracellular cholesterol production, respectively. The marked apoptotic effect observed by 2-DG treatment is not surprising since glucose addiction is a hallmark of aggressive tumor cells [72]. However, it should be emphasized that 2-DG treatment not only reproduces acute glucose deprivation, but it also interferes with N-linked glycosylation processes [73]. In a panel of ARMS cell lines, 2-DG was reported to induce cell apoptosis not by ATP loss but mainly through endoplasmic reticulum stress [74]. Therefore, further work is needed to characterize the mechanisms of toxicity induced by 2-DG treatments in myrAkt1 cells. In addition to 2-DG, the cytotoxic effects of lovastatin on myrAkt1 cells were particularly relevant, suggesting an important contribution of the mevalonate pathway in myrAkt1-driven tumorigenesis. Interestingly, oncogenic Akt/mTOR signaling has been shown to promote fatty acid and cholesterol synthesis through activation of the sterol responsive element binding protein (SREBP) [47]. Dysregulation of the mevalonate pathway through HMGCR has been shown to promote transformation [75], and epidemiological studies have confirmed a positive association between elevated serum cholesterol levels and tumor risk [76]. Thus, statin efficacy has been reported in several types of tumors [77], including RMS [78]. The mevalonate pathway uses acetyl-CoA to produce sterols and isoprenoid metabolites by a large number of enzymes [79], many of which have been shown to be essential for tumor cell survival [80]. Among the isoprenoid metabolites, farnesyl-diphosphate and geranylgeranyl-diphosphate contain hydrophobic chains that are essential for the isoprenylation of proteins. As a result, the inhibition of HMGCR by statins triggers the depletion of these isoprenoid pools, potentially affecting the localization and function of many isoprenylated proteins, as occurs for small GTP-ases [81]. Furthermore, since highly proliferative tumor cells need to produce membranes rapidly and cholesterol is an integral component of lipid rafts, cholesterol lowering agents inhibit this process. Overall, our data suggest that deregulated Akt1/mTOR signaling in ERMS cells, promoting an aggressive tumor phenotype, can become the Achilles’ heel for the tumor if the availability of some nutrients or their metabolism is disrupted. Given the observed efficacy of 2-DG and lovastatin in reducing cell malignancy, their use could represent a treatment option in combination with chemo and radiotherapy to weaken tumor metabolism. However, it is important to remember that the experiments shown in this work were performed using a single ERMS cell line that ectopically expresses an activated form of Akt1, and therefore conclusions must be drawn with caution. In conclusion, the characterization of the complex framework of Akt substrates (over 100) is required to understand the mechanisms leading to increased tumorigenicity. For example, activation of the Akt pathway due to a FGFR4 mutation was recently found in one high-grade RMS in association with a RAB3IP-HMGA2 fusion gene rearrangement [82]. The HMGA2 protein is a non-histone chromatin factor involved in DNA repair [83], aggressiveness of esophageal squamous cell carcinoma [84], epithelial-to-mesenchymal transition (EMT) process [85,86], and the up-regulation of EMT-related genes such as TGF-beta, SNAIL1, SLUG, MMP2, and MMP9 in RMS patients [87]. In this regard, the myrAkt1 cell model can represent an effective tool to validate Akt downstream targets potentially involved in ERMS tumorigenesis.

## Data Availability

Not applicable.
